# Focused abdominal sonography for trauma in the clinical evaluation of children with blunt abdominal trauma

**DOI:** 10.1186/s13017-015-0021-x

**Published:** 2015-07-01

**Authors:** Offir Ben-Ishay, Mai Daoud, Zvi Peled, Eran Brauner, Hany Bahouth, Yoram Kluger

**Affiliations:** Department of General of Surgery, Division of Surgery Rambam Health Care Campus, 8 Ha’Aliyah St, Haifa, 35254 Israel

## Abstract

**Introduction:**

In pediatric care, the role of focused abdominal sonography in trauma (FAST) remains ill defined. The objective of this study was to assess the sensitivity and specificity of FAST for detecting free peritoneal fluid in children.

**Methods:**

The trauma registry of a single level I pediatric trauma center was queried for the results of FAST examination of consecutive pediatric (<18 years) blunt trauma patients over a period of 36 months, from January 2010 to December 2012. Demographics, type of injuries, FAST results, computerized tomography (CT) results, and operative findings were reviewed.

**Results:**

During the study period, 543 injured pediatric patients (mean age 8.2 ± 5 years) underwent FAST examinations. In 95 (17.5 %) FAST was positive for free peritoneal fluid. CT examination was performed in 219 (40.3 %) children. Positive FAST examination was confirmed by CT scan in 61/73 (83.6 %). CT detected intra-peritoneal fluid in 62/448 (13.8 %) of the patients with negative FAST results. These findings correspond to a sensitivity of 50 %, specificity of 88 %, positive predictive value (PPV) of 84 %, and a negative predictive value (NPV) of 58 %. In patients who had negative FAST results and no CT examination (302), no missed abdominal injury was detected on clinical ground. FAST examination in the young age group (<2 years) yielded lower sensitivity and specificity (36 and 78 % respectively) with a PPV of only 50 %.

**Conclusions:**

This study shows that although a positive FAST evaluation does not necessarily correlate with an IAI, a negative one strongly suggests the absence of an IAI, with a high NPV. These findings are emphasized in the analysis of the subgroup of children less than 2 years of age. FAST examination tempered with sound clinical judgment seems to be an effective tool to discriminate injured children in need of further imaging evaluation.

## Background

Focused abdominal sonography for trauma (FAST) was first described in the early 1970s as an adjunct for injured evaluation in the emergency department. FAST has demonstrated its advantages as an easily comprehended examination, which is performed quickly, entails no radiation dose and has a reasonable sensitivity and specificity in adults.

FAST was first abandoned soon after its emergence, only to resurface in the 1990’s [1, 2] For adults, the use of FAST rapidly flourished, and in 1999, 80 % of the level I adult trauma centers reported its routine use [3]. Fast is used to detect fluid in the Morison and splenorenal pouch, pelvis and around the pericard, recently eFAST (Extended FAST) was introduced and included also the evaluation of both hemithoraxes. FAST has not gained popularity among pediatric trauma care providers. A national survey published in 2009 revealed that only 15 % of pediatric trauma centers in the United States adopted FAST as part of a blunt abdominal injury assessment protocol, compared to 96 % of the adult centers [4]. Therefore, the body of evidence for the use of FAST in the pediatric population is limited and mostly extrapolated from studies in adults. Furthermore, data on the sensitivity and specificity of FAST in toddlers under the age of 2 years is particularly deficient. Negus et al. addressed this issue and emphasized the need for pediatric separate guidelines [5].

The use of FAST as a triage tool for further investigation is important in an effort to reduce unnecessary exposure to ionizing radiation. Menaker et al. showed the use of FAST increases with the physician’s suspicion for IAI (Intraabdominal injury), and in patients with low and medium risk for IAI the use of FAST decreased the use of abdominal computed tomography [6].

The purpose of the current study was to assess the sensitivity and specificity of FAST for detecting free peritoneal fluid and abdominal injury in the pediatric population, with a particular focus on toddlers.

## Methods

We performed a retrospective analysis of prospectively collected data of the trauma registry of a single level I pediatric trauma center. Patients under the age of 18 years who underwent FAST during the period January 2010 through December 2012 were identified. Results of FAST, CT scan and operative findings were collected from patients’ electronic files. The primary outcome measure was the presence of free fluid in the peritoneal cavity, confirmed by CT scan. Secondary outcome measures were intra-abdominal injury (IAI), confirmed by CT scan or at laparotomy. The absence of IAI was defined either by a normal CT scan in patients that underwent one, or a clinical follow-up in patients who did not. Sensitivity, specificity, accuracy, and positive and negative predictive values were calculated for the primary and secondary outcome measures. Further subgroup analysis was undertaken for patients ≤ 2 years of age.

### FAST technique

According to hospital protocol, FAST examination was performed upon admission for all patients who sustained blunt abdominal injury, regardless of their hemodynamic status. Initial FAST examination was performed by a radiology resident, who in our institution is a trauma team member. The examiner routinely evaluated the presence of free fluid in the hepato-renal, spleno-renal, pelvis, and pericardial spaces. Results are reported as positive or negative without any further interpretation of intra-abdominal injury. FAST was performed with a Sonosite Ultrasound Machine M-Turbo, (FujiFilm). Using a Micromax abdominal curved array transducer 2-5MHZ. During the time frame of the study no protocol existed regarding the use of CT scan in this cohort of patients, decisions regarding the patient’s management were taken by the trauma attending in charge of the case, patients would either go to the OR, further imaging modalities or observation. The decision was taken namely according to the mechanism of injury and associated injuries.

### CT technique

The CT was performed with a Siemens Somatom definition flash, 128 channels. Iodine based IV contrast material (Iomeron 300, Dexxon, Or akiva, Israel) was injected intravenously in all patients. Our abdominal CT trauma protocol include a first scan that is performed with a delay of 70 s and a second delayed scan (5 min) for nephrographic delination. All scans were evaluated by a radiology resident and further re-evaluated by a radiology attending.

## Results

During the study, 543 children with suspected blunt abdominal injury were evaluated with FAST examination. The mean age was 8.2 ± 5 years. Ninety- five (17.5 %) had a positive FAST examination. CT scan was performed in 219 (40.3 %). A total of 22 (4 %) patients had abdominal injuries: 11 (50 %) had splenic injuries, 11 (50 %) liver injuries and 3 (13.6 %) small bowel injuries. One patient (4.5 %) sustained renal trauma. Exploratory laparotomy was performed in 9 (1.7 %) patients. One (0.2 %) succumbed to a severe head injury. Indications for laparotomy were hemodynamic instability in 5 (55.6 %) patients, failure of non-operative management of a grade IV liver injury in 1 (11.1 %), and a CT finding that suggested small bowel injury in 3 (33.3 %) patients.

**Fig. 1:**
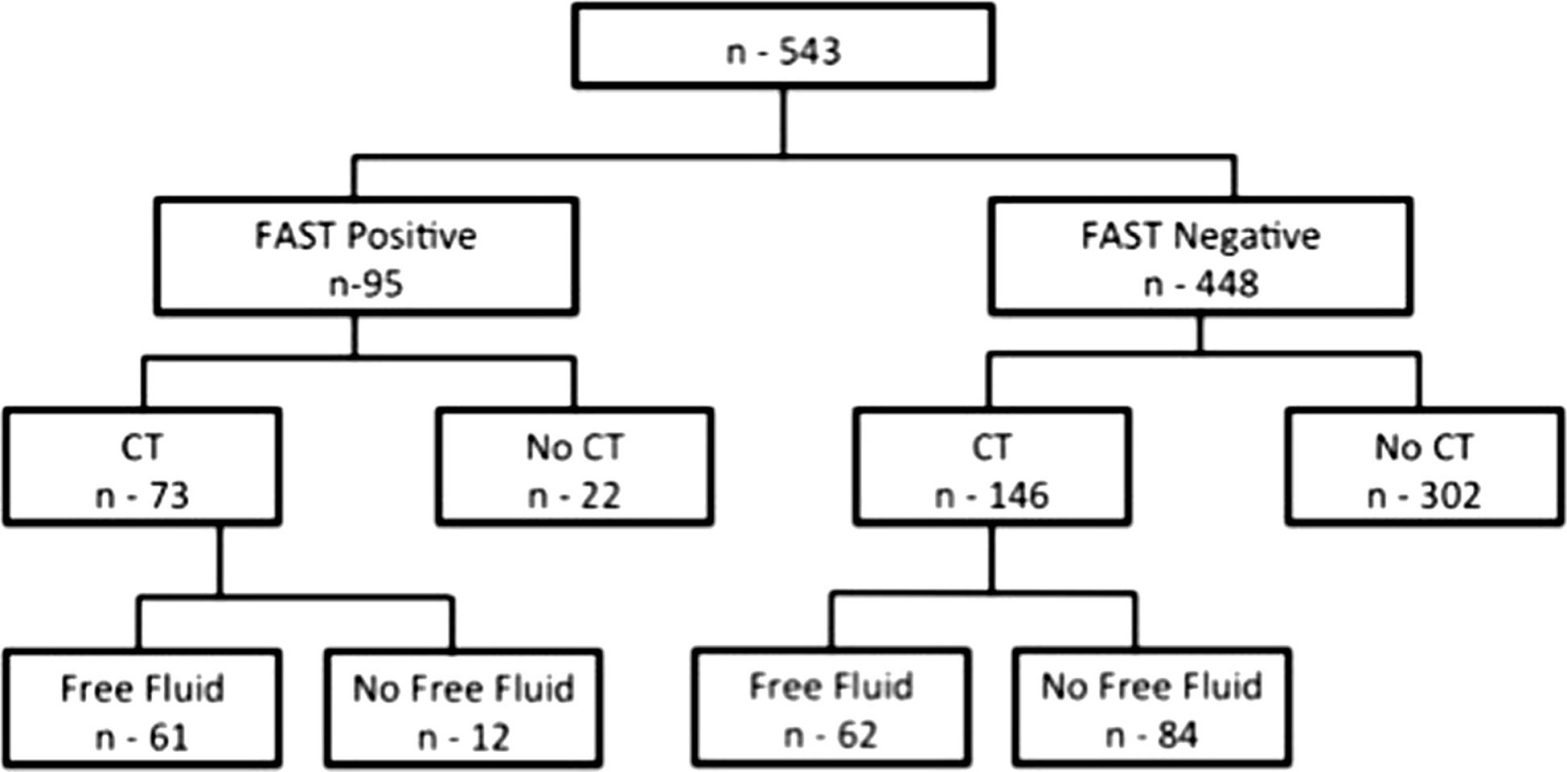
The detection of free fluid in children, according to FAST and CT results

Of the 95 patients with positive FAST results, CT was performed in 73 (76.8 %) and free fluid was detected in 61 (64.2 %) (Fig. 1). Thus, the use of FAST for the detection of free peritoneal fluid yielded a sensitivity of 50 %, specificity of 88 %, and a positive predictive value (PPV) of 84 % (Table 1).

**Table 1:** Sensitivity, specificity, PPV, NPV, and accuracy all of the groups

	Overall (n-543)		>2 years (n-454)		<2 years (n-89)	
	Free fluid	IAI^a^	Free fluid	IAI^a^	Free fluid	IAI^a^
Sensitivity	50 %	77 %	51 %	75 %	37 %	100 %
Specificity	88 %	70 %	90 %	69 %	78 %	72 %
PPV	84 %	22 %	88 %	22 %	50 %	20 %
NPV	58 %	97 %	56 %	96 %	67 %	100 %
Accuracy	66 %	66 %	64 %	70 %	62 %	74 %

*PPV* positive predictive value, *NPV* Negative Predictive value, *IAI* Intra-abdominal injury

^a^IAI: intra-abdominal injury

**Fig. 2:**
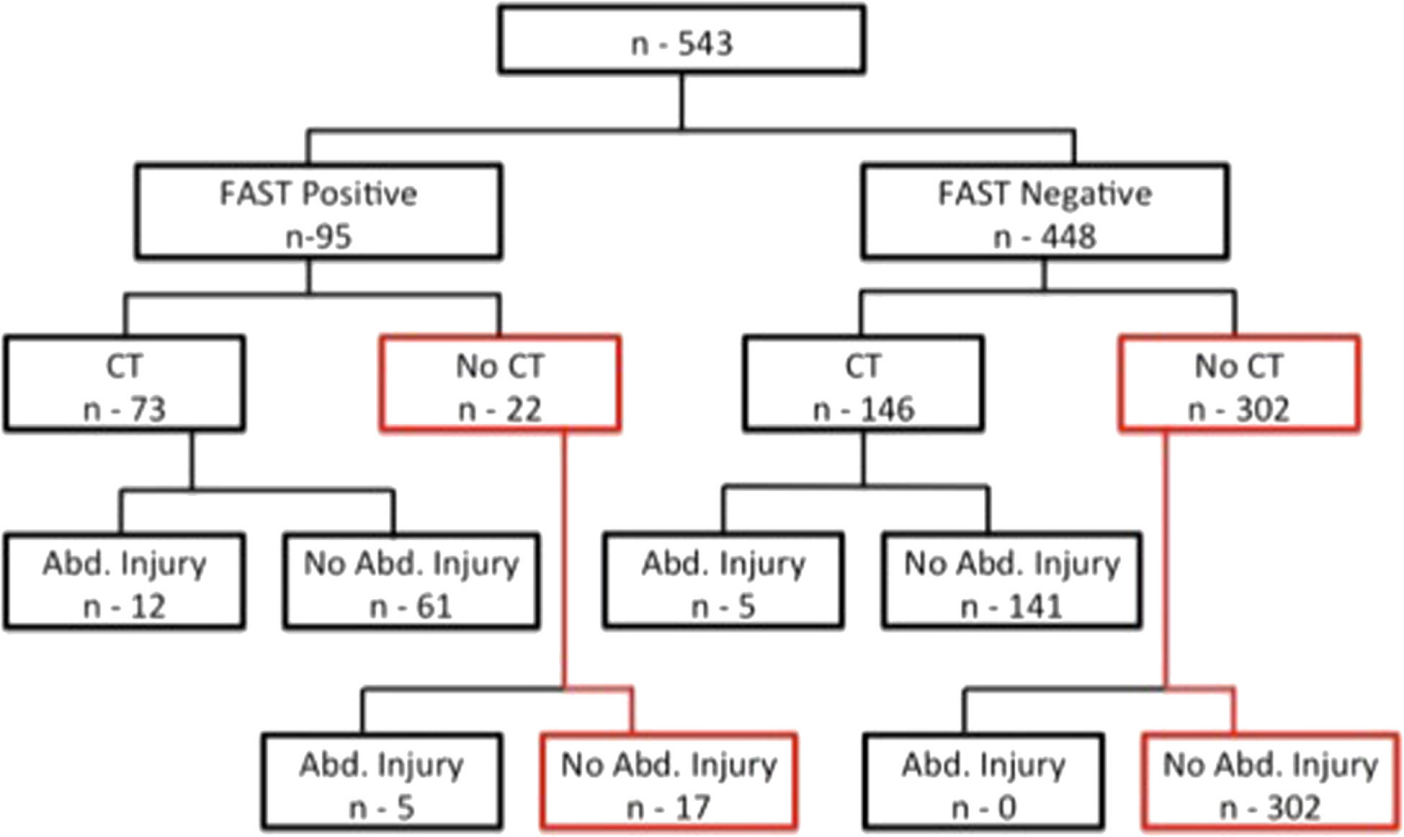
The detection of intra-abdominal injuries in children, according to FAST and CT results

Intra abdominal injury (IAI) was detected in 12 of the 73 patients who had positive FAST results and underwent CT (Fig. 2). None of the patients with a positive FAST result who did not undergo CT had a clinically significant missed IAI, based on clinical findings and follow-up of these patients. The 5 who had an IAI were operated based on the positivity of the FAST examination and their hemodynamic status. Thus, the detection by FAST of IAI yielded a sensitivity of 77 %, specificity of 70 %, and a negative predictive value (NPV) of 97 % (Table 1).

**Fig. 3:**
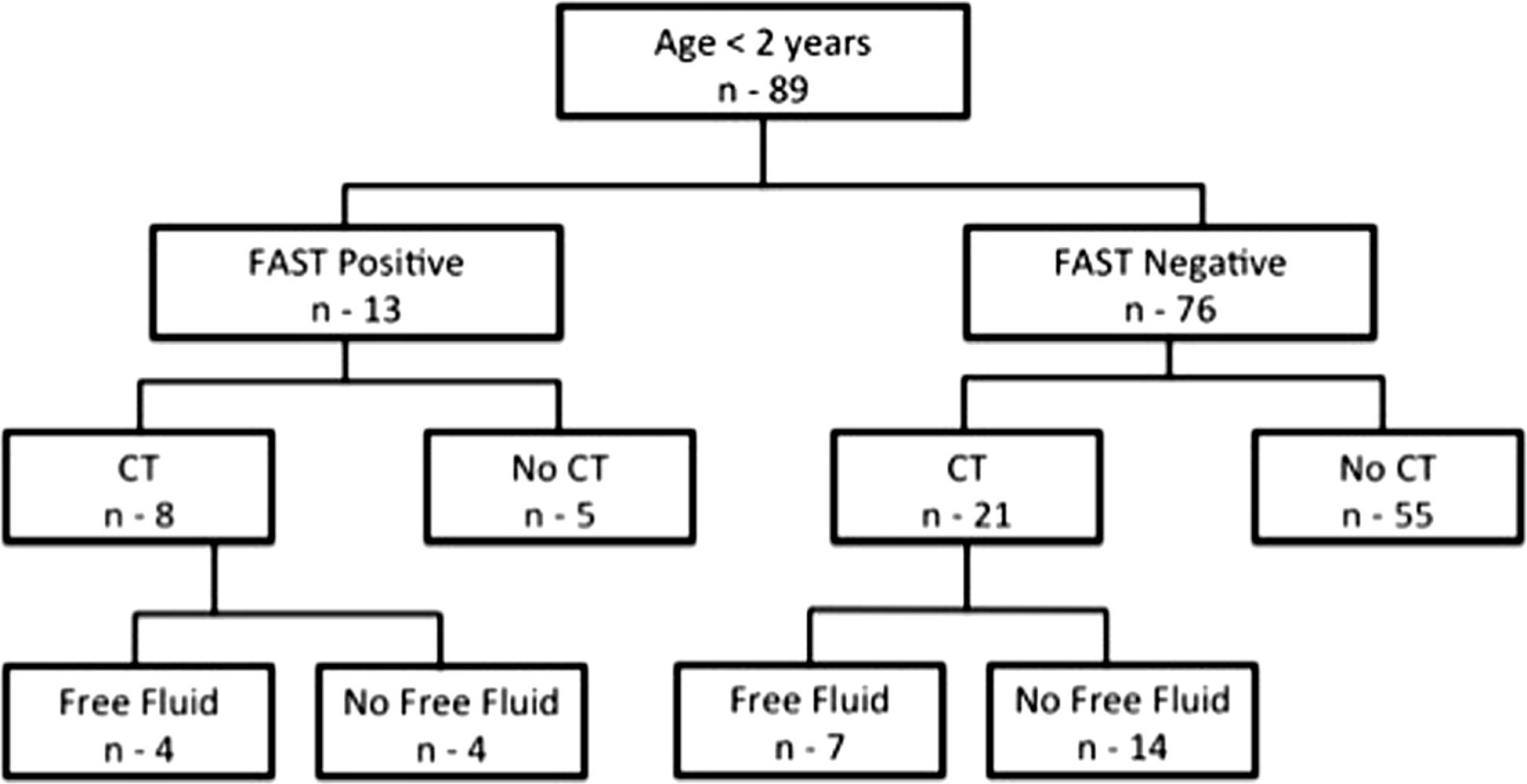
The detection of free fluid in children under the age of 2 years, according to FAST and CT results

**Fig. 4:**
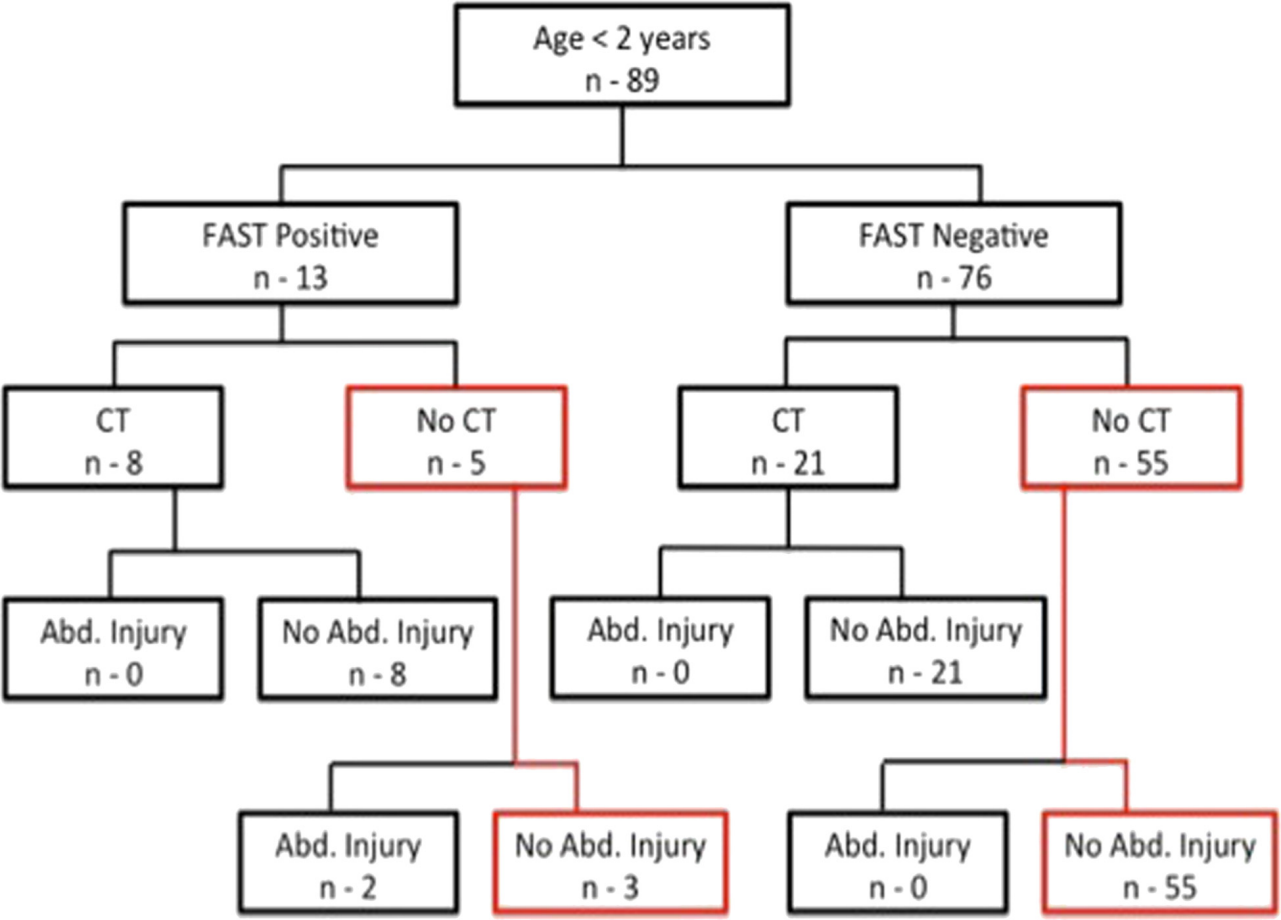
The detection of intra-abdominal injuries injuries in children under 2 years of age, according to FAST and CT results

In a subgroup analysis of the 89 (16.4 %) toddlers under the age of 2 years, 13 (14.6 %) had positive FAST results (Fig. 3). CT was performed in 8/13 (61.5 %) of them, and free fluid detected in 4/8 (50 %). Thus, the use of FAST for the detection of free peritoneal fluid in children aged < 2 years yielded a sensitivity of 37 % and a specificity of 78 %, with a fairly low PPV and NPV (50 and 67 % respectively). Two of the patients with a positive FAST result had an IAI (Fig. 4) and were transferred directly to the operating room due to hemodynamic instability. None of the 76 patients with a negative FAST had an IAI. Thus, the correlation of FAST with IAI yielded a sensitivity of 100 % and specificity of 72 % with a low PPV but a fairly high NPV (20 and 100 % respectively).

## Discussion

In recent years most adult trauma centers have integrated the FAST examination into an assessment protocol of blunt abdominal injury. However, pediatric trauma centers have responded tepidly to the incorporation of this technology. We believe that the main reason for the low adoption of FAST in the evaluation of children is the rare occurrence of unstable children with IAI. In adults FAST has almost eliminated the need for deep peritoneal lavage (DPL) that was used extensively in the past. Although rarely used DPL have the advantage of not only detecting free fluid in the abdominal cavity but also to elaborate on its quality (blood, bowel content, urine etc.).

Previous studies reported a wide range of sensitivity and specificity of the use of FAST in the pediatric population (30-97 % and 50-97 % respectively) [7–11]. The low sensitivity is partially due to the supposition that only one third of the children with IAI present without free fluid in the abdomen [12]. Furthermore, there is a severe paucity of evidence regarding the use of FAST in children younger than 2 years of age. The current study clarifies the contemporary use of FAST in a level I pediatric trauma center. We calculated sensitivity, specificity, accuracy, PPV, and NPV, not only for the presence of free fluid in the abdomen but also for the actual correlation IAI diagnosed either by CT scan or at laparotomy. No IAI was defined on clinical bases during the child’s admission and follow-up in the outpatient clinic.

Reported ranges of sensitivity and specificity for FAST in adults are: 73 - 88 % and 96 - 98 % respectively [13–15]. Our results are consistent with previous studies reporting low sensitivity (50 %) and reasonable specificity (88 %) of FAST in children [11]. Accuracy in the current study is significantly lower (66 %) than values reported for adults (96-98 %). Our results showed greater sensitivity and somewhat lower specificity (77 and 70 % respectively) for anticipating IAI than for the detection of free fluid. Although the presence of free fluid in the abdomen did not directly correlate with IAI (PPV - 22 %), the absence of fluid strongly suggests the absence of IAI (NPV - 97 %). These data contradict the previous assumption that one third of children with abdominal blunt trauma are without free fluid in the abdomen [12].

FAST was able to predict the need for an exploratory laparotomy in 89 % (*n* = 8) of the injured children in the current study. The only child who needed a laparotomy and had a negative FAST examination was a 16 year old with a handle bar injury. He presented with peritonitis of the upper abdomen. A CT scan showed a minimal amount of free fluid in the pelvis and a loop of small bowel with thickened wall and haziness of the fat around it. Therefore, he underwent an exploratory laparotomy that revealed small bowel injury. The ability to predict the need for laparotomy in our study is limited but the small number of patients who needed surgery all together.

In the subgroup analysis of children under age of 2 years, sensitivity and specificity for the presence of free fluid did not markedly differ from that of the whole cohort (37 and 78 % respectively). However, for IAI, sensitivity and NPV were both 100 % (Table 1). These findings suggest that FAST examination tempered with sound clinical judgment may reduce the need for further imaging and therefore reduce the radiation exposure of children under the age of 2 years.

In most centers in North America, the FAST examination is performed by a surgery resident. A number of studies showed equivalent accuracy when FAST is performed by surgeons, emergency medicine physicians, ultrasound technicians, and radiologists [16–19]. More recently, a Canadian survey showed that only 39 % of the surgical residents felt comfortable to make treatment decisions based on FAST examinations that they performed [20]. Although surgical residents are trained to perform FAST examinations in our institution, these exams are traditionally performed by radiology residents and subsequently evaluated by radiology attending physicians. The current study does not evaluate the differences between the two and therefor no conclusions could be extracted.

## Conclusions

This study shows that although a positive FAST evaluation does not necessarily correlate with an IAI, a negative one strongly suggests the absence of an IAI, with a high NPV. These findings are emphasized in the analysis of the subgroup of children less than 2 years of age. FAST appears to be best used for the detection of free fluid in the abdomen, as a surrogate of IAI in the unstable patient. It may be used as an adjunct in the assessment of the stable patient, to reduce the use of radiation, especially in children. Our findings support the integration of FAST into an assessment protocol of blunt abdominal injury in children. The prospective assessment of the impact of such protocol on the clinical outcome and the actual reduction of the use of unnecessary radiation emitting exams is needed.
